# Programmed Cell Death Progresses Differentially in Epidermal and Mesophyll Cells of Lily Petals

**DOI:** 10.1371/journal.pone.0143502

**Published:** 2015-11-25

**Authors:** Hiroko Mochizuki-Kawai, Tomoko Niki, Kenichi Shibuya, Kazuo Ichimura

**Affiliations:** NARO Institute of Floricultural Science, Tsukuba, 305–8519, Japan; Mediterranean Agronomic Institute at Chania, GREECE

## Abstract

In the petals of some species of flowers, programmed cell death (PCD) begins earlier in mesophyll cells than in epidermal cells. However, PCD progression in each cell type has not been characterized in detail. We separately constructed a time course of biochemical signs and expression patterns of PCD-associated genes in epidermal and mesophyll cells in *Lilium* cv. Yelloween petals. Before visible signs of senescence could be observed, we found signs of PCD, including DNA degradation and decreased protein content in mesophyll cells only. In these cells, the total proteinase activity increased on the day after anthesis. Within 3 days after anthesis, the protein content decreased by 61.8%, and 22.8% of mesophyll cells was lost. A second peak of proteinase activity was observed on day 6, and the number of mesophyll cells decreased again from days 4 to 7. These biochemical and morphological results suggest that PCD progressed in steps during flower life in the mesophyll cells. PCD began in epidermal cells on day 5, in temporal synchrony with the time course of visible senescence. In the mesophyll cells, the KDEL-tailed cysteine proteinase (*LoCYP*) and S1/P1 nuclease (*LoNUC*) genes were upregulated before petal wilting, earlier than in epidermal cells. In contrast, relative to that in the mesophyll cells, the expression of the SAG12 cysteine proteinase homolog (*LoSAG12*) drastically increased in epidermal cells in the final stage of senescence. These results suggest that multiple PCD-associated genes differentially contribute to the time lag of PCD progression between epidermal and mesophyll cells of lily petals.

## Introduction

Petal senescence is usually classified as a process of programmed cell death (PCD) in which cellular constituents are degraded and nutrients are remobilized from senescent tissues [1,2]. Expression of multiple genes encoding, such as nucleases, lipases, and proteinases, are involved in the process of PCD [2].

The total proteinase activity increases and the protein content decreases with tepal senescence in *Lilium*, *Iris*, and *Alstroemeria* flowers [3–6]. Inhibitor studies indicated that the majority of proteinase activity during petal senescence is because of cysteine-type proteinases [4,5]. In petunia petals, multiple genes of cysteine proteinases demonstrated different temporal expression patterns through development and aging [6]. Six of nine cysteine proteinase genes were found to be upregulated in the natural aging process, whereas three genes were highly expressed before visible symptoms of senescence were observed in petals and were downregulated in the senescent stage [6].

The senescence-associated cysteine proteinase SAG12 (senescence-associated gene 12) has been identified in *Arabidopsis* leaves [7]. Expression of SAG12 genes was limited to chloroplast-containing mesophyll and guard cells in the senescing leaves of *Arabidopsis* and soybean [8]. *SAG12* homologs cloned from petunia [6] and *Ipomoea nil* flowers [9] were upregulated in the senescent stage. However, the type of cells that mainly contain *SAG12* transcripts in petals is unknown.

KDEL-tailed cysteine proteinases also play an important role in plant PCD [10,11]. KDEL-tailed proteinases are synthesized as proenzymes with a C-terminal KDEL endoplasmic reticulum retention signal. When the C-terminal KDEL sequence is removed with the prosequence, the enzyme is activated [11]. In petals, KDEL-tailed cysteine proteinases are found in petunia [6], *Hemerocallis* [12], and *Lilium longiflorum* [10]. Transcript levels of *LlCYP*, the *L*. *longiflorum* KDEL proteinase gene, were low from bud development to full bloom but increased in the senescent stage [10]. In contrast, the *PhCP6* petunia KDEL proteinase gene was highly expressed in the early stage of flower life but was downregulated as senescence progressed [6].

Caspases are cysteine proteinases and important regulators of PCD in animal systems (e.g., [13]). *Arabidopsis thaliana* has four vacuolar processing enzyme (VPE) genes: *αVPE*, *βVPE*, *γVPE*, and *δVPE*, which encode enzymes with caspase-like activity. *βVPE* and *δVPE* are expressed in the seed and are involved in seed development [14,15]. *αVPE* and *γVPE* are preferentially expressed in vegetative tissues and are involved in PCD during leaf natural senescence and diverse stresses [15,16]. VPE was upregulated in senescent carnation petals [17], whereas in *I*. *nil* petals, the expression of the vegetative VPE homolog gradually increased during flower maturation and decreased in the senescent stage [18].

DNA degradation is widely observed during PCD in plant and animal cells. The putative nucleases *Hemerocallis DSA6* [19] and daffodil *DAFSAG1* [20] were upregulated with age-associated senescence in petals. The amino acid sequences of *DSA6* and *DAFSAG1* demonstrate similarity to S1- and/or P1-type nucleases. S1/P1 nucleases are the main class of enzyme involved in nucleic acid degradation in plant PCD [21].

Following PCD in petals, proteins, nucleic acids, and other macromolecules are degraded and transported from senescent tissues to developing parts for nutrient recycling [1,2]. Therefore, PCD in senescent petals appears to play an important role in nutrient recycling in plants.

In some species of petals, PCD begins earlier in mesophyll cells than in epidermal cells [22–24]. Our previous study revealed that DNA degradation and collapse of cells had begun in mesophyll cells before the petals (inner tepals) had visibly wilted, whereas, in tulip petals, DNA degradation and cell collapse was not observed in epidermal cells at that time [22]. However, little is known regarding the biochemical features of PCD in epidermal and mesophyll cell types in petal. Furthermore, the molecular mechanism underlying the PCD time-lag between different tissues in petals remains unclear.

In this study, we investigated the temporal and spatial differences of PCD progression during natural senescence in *Lilium* flowers by separately sampling epidermal and mesophyll cells from petals. *Lilium* is an important commercial cut flower and a useful model for studying PCD mechanisms in an ethylene-insensitive species. We performed morphological observations and compared biochemical PCD signs, including DNA degradation, protein content, proteinase activity, and expression patterns of genes encoding SAG12, KDEL-tailed cysteine proteinase, VPE, and S1/P1 nuclease in epidermal cells with those in mesophyll cells in *Lilium* petals. We discuss the contributions of PCD in epidermal and mesophyll cells to the recycling of nutrients during age-associated petal senescence.

## Materials and Methods

### Plant materials

Oriental-Trumpet hybrid lily (*Lilium* cv. Yelloween) flower stems were obtained from a commercial grower in Uonuma city, Niigata prefecture, Japan. The stems were harvested and sent to our laboratory on the same day. On the next day, we received the cut flower stems and placed them in distilled water (23°C, 70% relative humidity, 12-h photoperiod at 10 μmol m^−2^s^−1^ PPFD) until their anthers were dehiscent. Five to six buds were attached to each cut plant. We detached two or three flowers from the bottom part of each plant when their anthers were dehiscent (day 0). Individual cut flowers were placed in conical flasks containing 300-mL distilled water and held as described above until petal abscission.

In biochemical and molecular biological analyses, tweezers were used to drag a layer of sample adaxial and abaxial petal (inner tepal) epidermis away from the petal mesophyll [22]. The separated adaxial epidermal layer and the remaining mesophyll cells, including mesophyll and vascular cells, were sampled separately. Central bundles and the bottom part of each petal were excluded from petal samples. Each sample was frozen in liquid N_2_ and stored at −80°C until use.

### Measurement of petal fresh and dry weight

We investigated the life span of petals from 0 to 7 days after anthesis. To determine the fresh weight (FW) of whole petals, we sampled petals from three flowers at each time point, and recorded the fresh weight of each sample. Dry weight was measured after samples were dried in an electric oven at 80°C for 48 h. To determine the dry weight of epidermal and mesophyll cells per petal, we sampled each type of cells from three flowers at 0, 2, 4 and 6 days after anthesis.

### Light microscopy observation and viability staining test

At each time point, a petal was taken from each of the three flowers, and a 2 × 5-mm section was cut from the central part of each petal. The sections were fixed in FAA solution [formalin, acetic acid, 80% ethanol, 1:1:8 (v/v)]. The samples were dehydrated in a series of graded ethanol solutions and embedded in Technovit 7100 (Heraeus Kulzer, Wehrheim, Germany). Sections (10 μm) were cut with a microtome and stained with toluidine blue dye. A light microscope (Olympus AX70, Olympus, Tokyo, Japan) was used for all histological observations. We randomly selected three areas (1-mm length) from each section and separately counted the unbroken epidermal and mesophyll cells. The experiments were performed in triplicate with independent samples.

For a viability staining test, we used Evans Blue dye (Wako, Osaka, Japan) bound to dead cells. We cut a 3 × 20-mm section from the central part of each petal at 0, 2, 4, and 8 days after anthesis. The sections were completely submerged in 0.1% (w/v) aqueous Evans Blue dye and incubated for 90 min at room temperature (25°C). They were then washed twice (30 s each) with distilled water at room temperature to remove unbound dye. To observe the epidermal and mesophyll tissues separately, the adaxial epidermal layer dragged away from each section with tweezers. The experiments were performed in triplicate with independent samples.

### Protein content and total proteinase activity

Protein extraction and proteolytic activity assays followed the protocol described by Battelli et al. [3]. The Qubit Protein Assay kit (Invitrogen, Carlsbad, CA, USA) was used according to the manufacturer’s instructions to determine the soluble protein content. The proteolytic activity was assayed at pH 5.5 in 500-μL reaction mixtures (22.5 mM of acetate buffer (pH 5.5), 0.2% (w/v) azocasein, 2.5 mM β-mercaptoethanol) and 20-μL extract [3]. Each mixture was incubated at 32°C for 16 h and the enzymatic reactions were stopped by the addition of 125 μL 50% trichloroacetic acid (TCA) (w/v). Reaction mixtures were centrifuged at 14,000 × *g* for 10 min and absorbance at 330 nm was measured. These measurements were performed in triplicate with independent samples.

### DNA degradation

Total DNA was extracted from each sample using a modified cetyltrimethylammonium bromide (CTAB) method [25]. Each DNA sample (500 ng) was subjected to electrophoresis in a 3% (w/v) agarose gel, and gels were stained with GelRed (Biotium, Inc., USA). The experiments were performed in triplicate with independent samples.

### Isolation of total RNA and synthesis of cDNA

For isolation of RNA, each sample (100 mg) was frozen in liquid nitrogen and ground into powder in a 2-ml microtube. Total RNA was extracted from the flowers with an RNeasy Plant Mini Kit (Qiagen, Hilden, Germany) after RNase-free DNase І (Qiagen) treatment. Between 0.1 and 0.5 μg of the total RNA was used to synthesize first-strand cDNA with oligo-dT primer and random 6-mers using reverse transcriptase with a PrimeScript RT master mix kit (Takara Bio, Otsu, Japan) according to the manufacturer's instructions.

### Cloning of cDNA

Degenerate primers for amplification were designed on the basis of sequences corresponding to highly conserved peptide regions of SAG12-cystein proteinase and S1/P1-type nuclease. To clone VPE, KDEL-tailed cysteine proteinase, and ubiquitin (UBQ), specific primers were designed using the cDNA sequences of *LlVPE4*, *LlVPE5*, *LlCYP* [10,26], and *UBQ* (AF116772) identified from *L*. *longiflorum*. cDNAs were synthesized with RNA extracted from petals on days 2 and 6. Amplified polymerase chain reaction (PCR) products of appropriate length were subcloned into the pGEM-T-easy vector (Promega, Madison, WI, USA) and sequenced with a Big Dye Terminator v3.1 Cycle Sequencing Kit (Applied Biosystems, Foster City, CA, USA) on the ABI PRISM 3100 Genetic Analyzer (Applied Biosystems). We obtained full-length composite cDNA (S1P1 type nuclease, SAG12, KDEL, VPE, and UBQ) by 3′- and 5′-rapid amplification of cDNA ends (RACE). Primers were designed using Primer3 (http://bioinfo.ut.ee/primer3-0.4.0/) on the basis of the resulting DNA sequences. RACE products were cloned into the pGEM-T-easy vector (Promega) and sequenced as described above. For further cloning of the full-length cDNAs, PCR was performed using gene-specific primers with the Prime STAR GXL DNA polymerase (Takara Bio) according to the manufacturer's instructions.

### Quantitative real-time RT-PCR analysis

Total RNA isolation and synthesis of cDNA were performed as described above. The primers for real-time RT PCR were designed to target the 3′-untranslated region of target genes with Primer3 (S1 Table). PCR reactions were performed using SYBR Premix Ex Taq Π (Takara Bio) on a Thermal Cycler Dice Real Time System (TP600, Takara Bio). The thermal cycling conditions were 95°C for 10 s followed by 40 cycles of 95°C for 5 s and 60°C for 30 s. Data were normalized by the calculation of the transcript level ratios of four target genes and UBQ within the same sample. These measurements were performed in triplicate with independent samples.

### Nitrogen analysis

Tissues were dried in an electric oven at 80°C for 24 h. For nitrogen (N) analysis, each dried sample was ground with a pestle and mortar. Determination of total N content followed the protocol described by Makino et al. [27]. Briefly, the dried ground materials were digested with H_2_SO_4_–H_2_O_2_ and total N content was determined with Nessler’s reagent [27]. These measurements were performed in triplicate with independent samples.

## Results

### Time course of flower senescence

**Fig 1:**
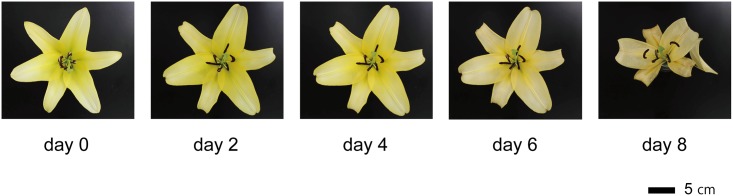
Stages of *Lilium* cv. Yelloween flower lifespan from flower opening to senescing petals.

**Fig 2:**
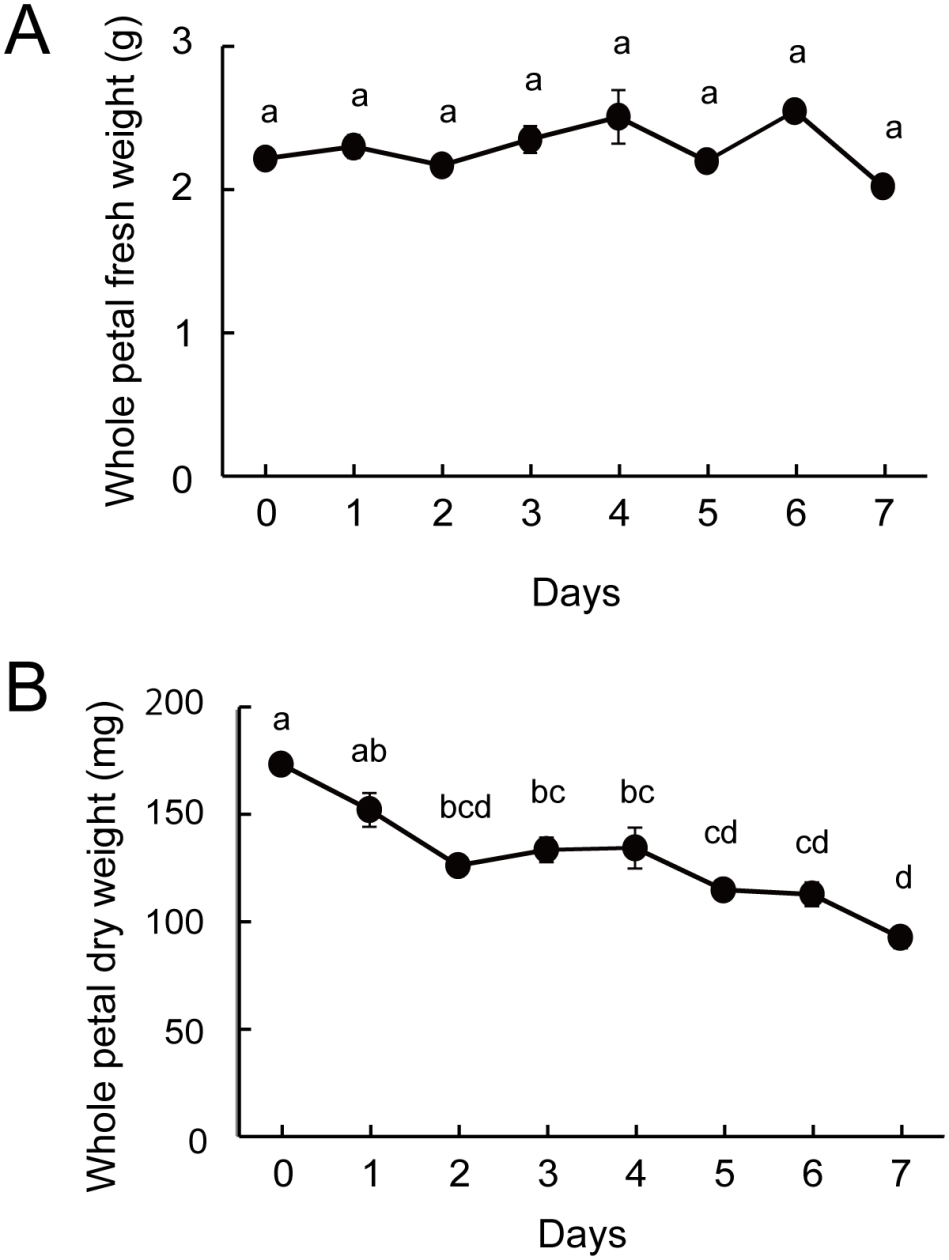
(A) Fresh weight and (B) dry weight of individual whole petals during flower development and senescence. Means with different letters are significantly different (Tukey test, *p* < 0.05). Each data point represents the mean ± S.E. (n = 3).

Visible signs of senescence, e.g., color fading in petals, began to be observed on day 5 and were fully evident on day 6. The tips of petals (inner tepals) were wilted on day 7, and petals abscised on day 8 (Fig 1). The FW of whole petals did not show apparent changes until abscission (Fig 2A). Dry weight of whole petals decreased in two steps: in the early (from day 0 to 2) and late (from day 4 to 7) stages of the experiment (Fig 2B, *p* < 0.05, Tukey test).

Dry weight began to decrease earlier in the mesophyll cells than in the epidermal cells (S1 Fig). The dry weight of the mesophyll significantly decreased in two steps: from days 0 to 2 and from days 4 to 6 (S1 Fig, *p* < 0.05, Tukey test). In contrast, the dry weight of the epidermal cells significantly increased from days 2 to 4 and decreased from days 4 to 6 (S1 Fig, *p* < 0.05, Tukey test).

### Light microscopy observations, change in cell number, and viability test

**Fig 3:**
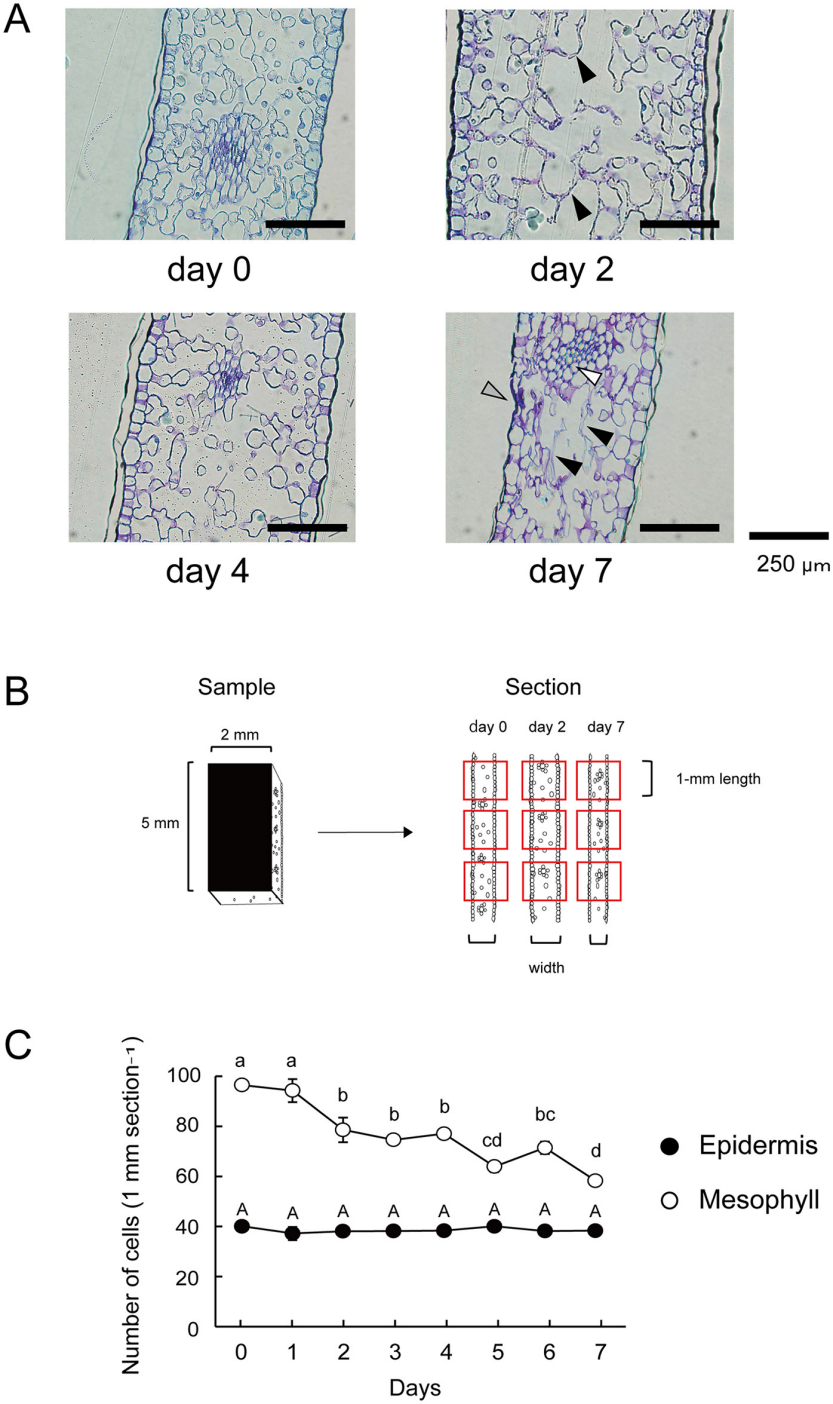
**(A) Cross sections of the middle part of the margin of *Lilium* cv. Yelloween petals.** Black arrows, collapsed mesophyll cells (day 2 and 7); white arrow, vascular strand cells (day 7); gray arrow, partially disrupted epidermal cells (day 7). Bars, 250 μm. **(B) Areas for counting the number of cells.** A 2 × 5-mm section was cut from the central part of each petal. Three areas were randomly selected from each section (red square) and unbroken epidermal and mesophyll cells were counted separately. The length of areas was fixed (1 mm), whereas widths of areas changed owing to the change in petal thickness during development and senescence. **(C) Changes in cell numbers in the epidermal and mesophyll layers.** Means with different letters are significantly different (Tukey test, *p* < 0.05). Each data point represents the mean ± S.E. (n = 9).

Images of cross sections prepared from the middle parts of petals are shown in Fig 3. On day 0, both epidermal and mesophyll cells were well defined (Fig 3A, day 0). However, in the mesophyll layer, some mesophyll cells collapsed and lost their organization at day 2 (Fig 3A, black arrows, day 2). On day 4, relative to the day 0 section, the intercellular spaces were widened in the mesophyll layer, but disruption of the cells was not evident (Fig 3A, day 4). The epidermis remained intact on day 4 (Fig 3A, day 4). On days 6 and 7, disrupted mesophyll cells were observed once again (Fig 3A, black arrows, day 7), but cells belonging to the vascular strand remained intact (Fig 3A, white arrow, day 7). The epidermal cells were partially collapsed at day 7 (Fig 3A, gray arrow, day 7).

The unbroken epidermal and mesophyll cells were counted in a 1-mm length of areas of each section. The thickness of petals changed through development and senescence, as did the width of petal sections. Thus, the length (1 mm) of the areas was fixed, whereas their width changed (Fig 3B). The number of epidermal cells did not change throughout the experiment, whereas the number of mesophyll cells decreased significantly in two steps: in the early (from day 1 to 2) and late (from day 4 to 7) stages of the experiment (Fig 3C, *p* < 0.05, Tukey test). The number of mesophyll cells decreased by 22.8% within 3 days after anthesis (Fig 3C).

In the viability test, the epidermal sections were not evidently stained until day 8 (S2 Fig, black arrow, day 8). The mesophyll tissues were stained only in a small part of the section on day 0. On days 2 and 4, in the mesophyll tissues, the sections were widely stained, and the stained area increased from day 4 to 8 (S2 Fig).

### Total proteinase activity, total protein content, and DNA degradation

**Fig 4:**
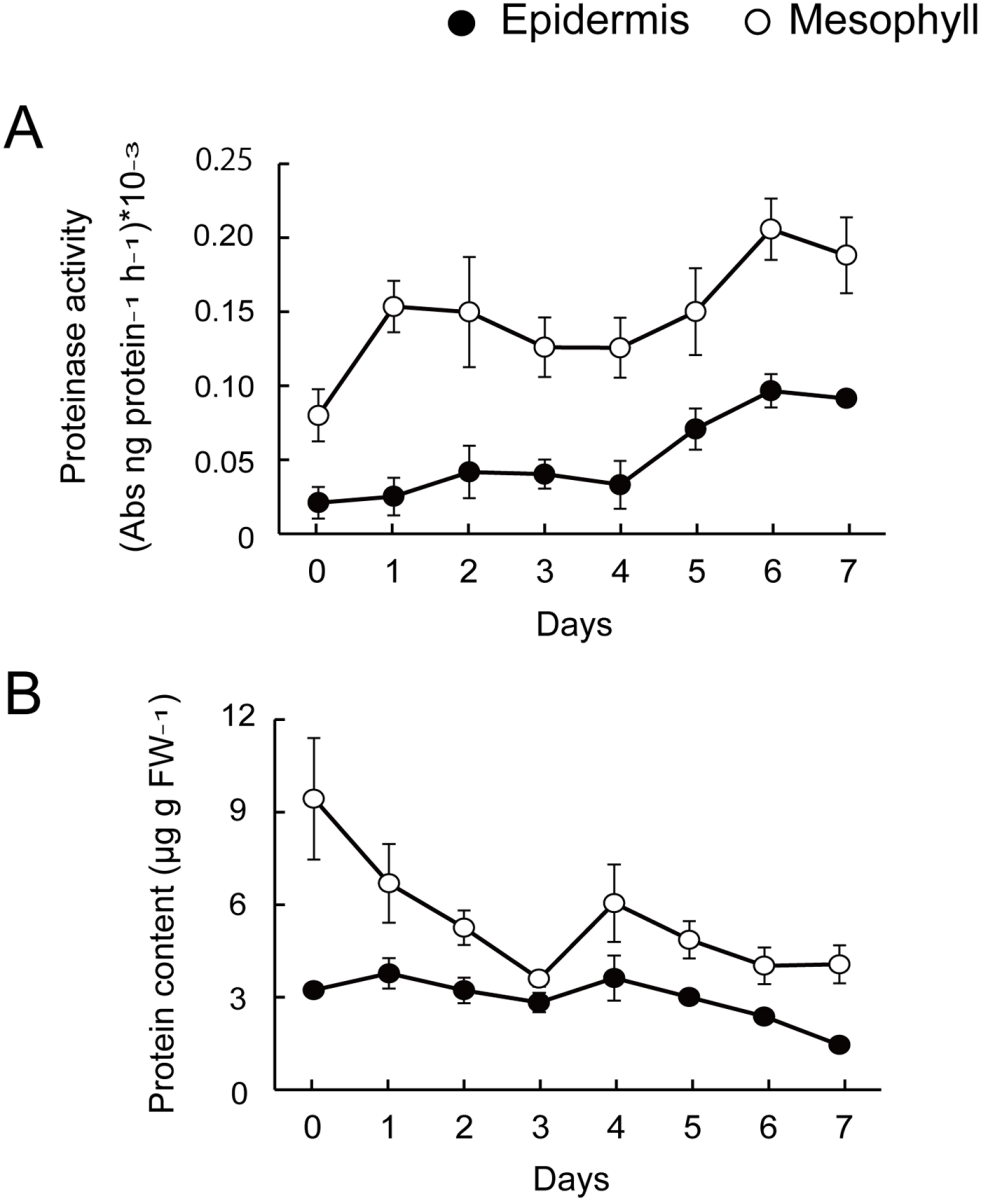
(A) Total proteinase activity and (B) protein content in *Lilium* cv. Yelloween petals. Each data point represents the mean ± S.E. (n = 3).

In epidermal cells, the proteinase activity increased from day 4 (Fig 4A). In contrast, mesophyll cell samples showed high proteinase activity on day 1 and a second peak of activity on day 6 (Fig 4A).

The soluble protein content decreased in the late stage (from day 4 to 7) in the epidermal cell samples (Fig 4B), consistent with the change in proteinase activity in the epidermis (Fig 4A). In the mesophyll cell samples, the soluble protein content began to decrease from day 0 (Fig 4B). Soluble protein content of mesophyll cells decreased by 61.8% within 3 days after anthesis (Fig 4B).

**Fig 5:**
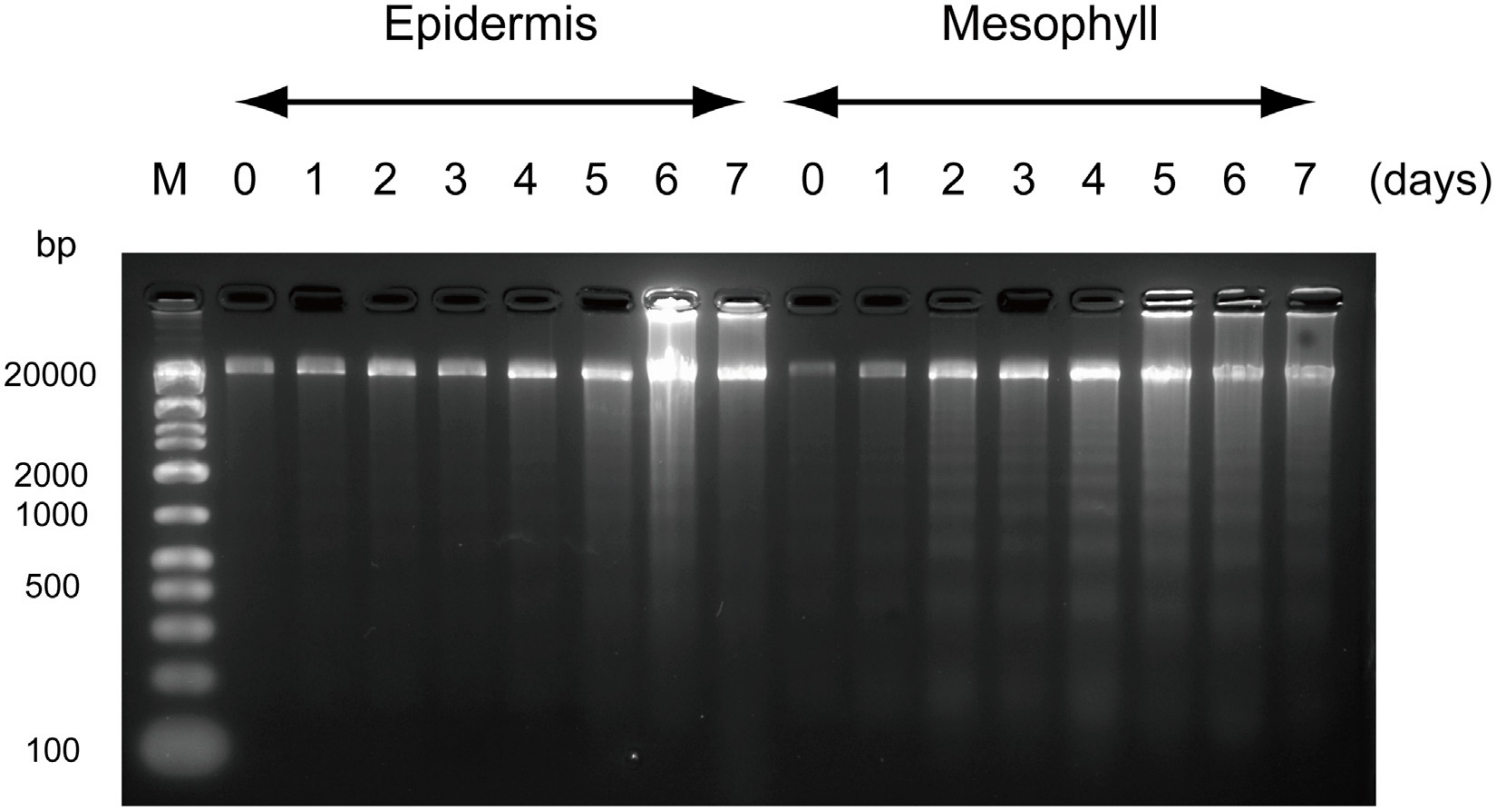
(A) DNA fragmentation at different stages of *Lilium* cv. Yelloween petal development from flower opening to senescence. M: DNA ladder marker.

In the epidermal cell samples, clear DNA degradation was observed on day 5 (Fig 5, left). In the mesophyll cell samples, a DNA laddering pattern was observed from day 2 (Fig 5, right) and gradually became marked during the development and senescence of flowers.

### Different expression patterns of PCD-associated genes

Three cysteine proteinase gene homologs, encoding SAG12, KDEL, and VPE, were isolated and designated as *LoSAG12*, *LoCYP*, and *LoVPE*, respectively (S3, S4 and S5 Figs).

The deduced amino acid sequence of *LoSAG12* showed 55–68% identity with sequences of SAG12 cysteine proteinases obtained from petals of petunia (AY662996) and leaves of *A*. *thaliana* (AAC49135) and *Nicotiana tabacum* (AY881011). Catalytic residues Cys-140, His-277, Asn-298, and several other characteristic amino acids are conserved in *LoSAG12* [28−30] (S3 Fig).

The deduced amino acid sequence of *LoCYP* showed 70–93% identity with sequences of KDEL-tailed cysteine proteinases cloned from petals of *L*. *longiflorum* (HF968474), *Hemerocallis* (U12637, X74406), and *Iris* (AY504967). The *LoCYP* protein carries a KDEL motif at the C terminus (S4 Fig), which acts as an ER-retention/retrieval signal and identifies *LoCYP* as a member of the KDEL-tailed cysteine proteinase family. The catalytic residues Cys-154, His-289, Asn- 310, and Gln-148 are conserved in *LoCYP* [3,28,29] (S4 Fig).

The deduced amino acid sequence of *LoVPE* showed 99% and 90% identity with the sequences of *LlVPE4* and *LlVPE5*, respectively, which were obtained from *L*. *longiflorum* [26] (S6 Fig). In phylogenetic analysis, *LoVPE* grouped in a large cluster of vegetative VPEs (S6 Fig). The deduced amino acid sequence of *LoVPE* showed higher identity with sequences of *Arabidopsis* vegetative αVPE (D61393) and γVPE (BAA18924) than with sequences of seed-type βVPE (D61394) and δVPE (AF521661). The catalytic residues Cys-204 and His-162 [15,26], and the C-terminal GFSA motif are conserved in *LoVPE* [26] (S5 Fig).

A homolog of S1/P1 type nucleases (*LoNUC*) was also isolated. The deduced amino acid sequence of *LoNUC* showed 70%–78% identity with the sequences of S1/P1 type nuclease genes from *Hemerocallis* (AF082031), *A*. *thaliana* (NM_100991.2), and *Zinnia elegans* (AB003131) (S7 Fig). The active-site residues responsible for the binding of zinc atoms are conserved in *LoNUC* [21,31] (S7 Fig).

**Fig 6:**
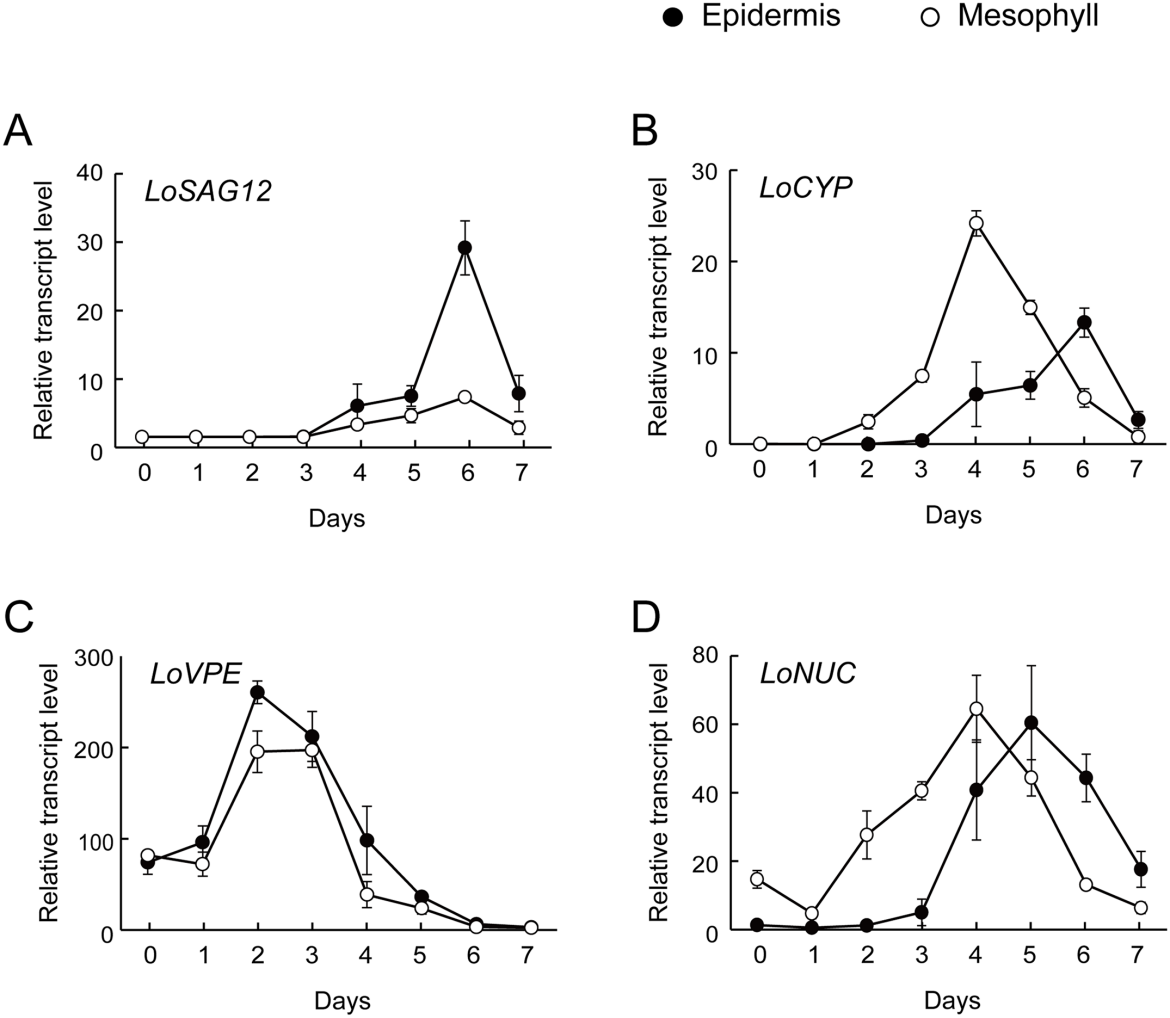
Transcripts of (A) *LoSAG12*, (B) *LoCYP*, (C) *LoVPE*, and (D) *LoNUC* homologs in petals of *Lilium* cv. Yelloween flowers. Values for each homolog are relative to the expression level of ubiquitin. Each data point represents the mean ± S.E. (n = 3).

Three cysteine proteinase homologs (*LoSAG12*, *LoCYP*, *LoVPE*) showed expression patterns differing between the epidermal and mesophyll cells during senescence (Fig 6A–6C). In both the epidermal and mesophyll cell samples, *LoSAG12* was upregulated in the senescent stage (Fig 6A). The peak transcript level of *LoSAG12* was higher in the epidermal cells than that in the mesophyll cells on day 6 (Fig 6A). By contrast, the transcript levels of the KDEL-tailed cysteine proteinase homolog (*LoCYP*) began to increase in mesophyll 2 days earlier than that in epidermis, and the transcripts peaked on day 4 (Fig 6B). In the epidermal cells, the peak of *LoCYP* transcripts appeared on day 6 (Fig 6B). Similar patterns were found in the epidermal and mesophyll cells in the levels of *LoVPE* homolog. The transcript levels of *LoVPE* increased on day 2 and decreased on day 4 (Fig 6C).

On day 0, the S1/P1 nuclease homolog (*LoNUC*) was detected in the mesophyll cells, whereas, in the epidermal cells, the transcript level of *LoNUC* was negligible. The transcript levels of *LoNUC* in the mesophyll cells began to increase on day 2, earlier than those in the epidermis (Fig 6D). The transcripts of *LoNUC* peaked on day 4 in the mesophyll cells, and on day 5 in the epidermal cells (Fig 6D). These peak levels of *LoNUC* transcripts were nearly identical in epidermal and mesophyll cells.

### Nitrogen decrease during senescence

In the epidermal cells, the nitrogen content did not show a significant decrease throughout the experiment (Table 1). By contrast, in the mesophyll cells, the nitrogen content decreased significantly from day 4 to day 7 (Table 1, *p* < 0.01, Tukey test).

**Table 1:** Changes in nitrogen content of epidermal and mesophyll cell tissues per petal from flower opening to senescence.

	Epidermal	Mesophyll
Day 0	20.4 ± 1.5 a	235.5 ± 6.8 a
Day 4	24.0 ± 1.4 a	226.4 ± 0.9 a
Day 7	16.8 ± 2.4 a	170.6 ± 6.3 b

Central bundles and the bottom part of each petal were excluded from samples. Means with different letters are significantly different in each tissue (Tukey test, *p* < 0.01). Each data point represents the mean ± S.E. (n = 3).

## Discussion

In this study, we confirmed that in petals of cut lilies, PCD began earlier in the mesophyll than in the epidermis, and before visible signs of senescence were observed. In mesophyll cells, DNA laddering, decreases in dry weight, protein content, an increase in proteinase activity, and tissues stained with Evans Blue dye were observed within 3 days after anthesis, and 22.8% of the cells were lost. The proteinase activity showed a second peak on day 6 and the number of mesophyll cells decreased again from days 4 to 7. These biochemical and morphological results suggest that, in the mesophyll cells of *Lilium* cv. Yelloween petals, PCD progressed in steps: in the early (from day 1 to day 3) and late (from day 5 to day 7) stages of flower life. The first onset of PCD appeared to cause early senescence in the mesophyll cells before wilting in petals. Although the mesophyll contained mesophyll and vascular strand cells, the present biochemical results likely reflect PCD in mesophyll cells rather than in vascular strand cells, given that vascular strand cells remained intact on day 7, according to light microscopy observations. In the epidermal cells, PCD progressed in one step. Signs of PCD, such as DNA laddering and decreases in protein content, appeared from 5 days after anthesis.

PCD-associated genes expressed in the epidermal and mesophyll cells in lily petals showed spatial and temporal expression differences. The present results suggest that in the mesophyll cells of *Lilium* cv. Yelloween petals, *LoCYP* and *LoNUC* genes are involved in the early onset of PCD during the early stages of flower life, and may facilitate the transportation of degraded nutrients from mesophyll cells. In the epidermal cells, *LoSAG12*, *LoCYP*, and *LoNUC* genes contribute to the late onset of PCD during senescence.

### Time course of PCD progression in epidermal and mesophyll cells

The results of our biochemical and morphological observations are consistent with those of previous studies showing that mesophyll cells begin to senesce earlier than epidermal cells in petals [22–24]. We also found a step-like progression of PCD in the mesophyll cells, which is expected to affect the reduction of dry weight in whole petals. This weight decreased twice during senescence. Our results are similar to those for the proteinase activity in whole tepals of *L*. *longiflorum* [3]. Those levels increased slightly from bud development to full bloom and increased sharply in the tepal senescent stage [3]. The present procedure, investigating epidermal and mesophyll cells separately, allowed us to identify an early increase in proteinase activity only in the mesophyll and not in the epidermal cells. Although the epidermal and mesophyll cells were not distinguished, a previous study found that 50% of nuclei showed TUNEL-positive signs of DNA fragmentation in the blooming stage in *L*. *longiflorum* [3]. The previous and present biochemical results suggest that PCD occurs in some mesophyll cells but not in all cells simultaneously during the early stages of flower life. Five days after anthesis, signs of PCD were found in both epidermal and mesophyll cells. The late onset of PCD was temporally synchronized with the course of visible senescence. In petals of *Lilium* cv. Yelloween, the first visible sign of senescence was color fading in the petals [32]. In *Lilium* cv. Yelloween, the petals contain carotenoid pigments, which are abundant in epidermal cells (data not shown). The progression of PCD in epidermal cells may trigger a decrease of carotenoid content in petals and induce color fading in *Lilium* cv. Yelloween [32].

### Different expression patterns of PCD-associated genes

The temporal expression pattern of *LoSAG12* was consistent with that of petunia and *I*. *nil* SAG12, which were upregulated in the senescent stage of the flowers [6,9]. However, the spatial expression pattern of *LoSAG12* differed from that of *Arabidopsis* and soybean SAG12 genes. *LoSAG12* was actively expressed in epidermis rather than mesophyll cells, whereas *Arabidopsis* and soybean SAG12 genes were expressed in mesophyll and guard cells but not in epidermal cells [8]. The spatial expression patterns of SAG12 may differ between petals and leaves. *Arabidopsis* and soybean SAG12 genes were expressed in chloroplast-containing leaf cells [8] and, in this study, *LoSAG12* was actively expressed in carotenoid-containing petal cells. These expression patterns imply that the SAG12 gene may be expressed in associated with the degradation of plastids during plant senescence. In *Lilium* cv. Yelloween, SAG 12 may degrade cellular components, including carotenoids in epidermal cells, possibly leading to color fading in petals.

From day 2, *LoCYP* was upregulated in the mesophyll cell samples. *LoCYP* may be involved in the early onset of PCD in mesophyll cells. The expression pattern of *LoCYP* was consistent with the results of previous studies with *Hemerocallis* and petunia flowers, in which KDEL-tailed cysteine proteinase homologs were expressed from the early stages of flower life [6,12]. An immunoreactive study found that the levels of proactive and active KDEL enzyme increased during flower opening as well as senescence [10]. These results suggest that KDEL-tailed cysteine proteinase is involved in early onset of PCD in mesophyll cells. The *LoCYP* gene was also upregulated in epidermal cells in the final stage of flower life, possibly leading to PCD in epidermal cells.

There were no differences in the *LoVPE* expression patterns between the epidermal and mesophyll cells, leaving the time lag of PCD progression between epidermal and mesophyll cells unexplained. The transcript level of *LoVPE* increased before petal wilting and decreased with age-related petal senescence. Our results are similar to those for *I*. *nil* petals, in which γVPE transcript levels increased before petal wilting and decreased in the senescent stage [18]. In the mesophyll cells, the total proteinase activity was high at day 0, prompting further studies to clarify the contributions of proteinases other than SAG12, KDEL, and VPE cysteine proteinases. Other types of cysteine proteinase, or aspartic, serine, and metalloproteinases, may contribute to proteinase activities in mesophyll cells.

On day 2, DNA degradation was observed, and expression of the S1/P1 nuclease homolog (*LoNUC*) was upregulated only in the mesophyll cells and not in the epidermal cells. In *Hemerocallis*, expression of the S1/P1 nuclease (*DSA6*) homolog began to increase before flower opening [19]. These results suggest that the S1/P1 type nuclease is involved in early PCD progression in mesophyll cells and contributes to the time lag of PCD between epidermal and mesophyll cells.

### Contributions to nitrogen remobilization

Dry weight decreased in mesophyll cells during senescence, suggesting that degenerated cellular components may be remobilized from senescent mesophyll cells. Although protein content decreased markedly during the early stage of senescence, nitrogen content decreased significantly during the later stage of senescence in mesophyll cells. Thus, nitrogenous compounds derived from the partial degradation of proteins appear to be remobilized from mesophyll cells during the later stage of senescence. In the epidermal cells, the nitrogen content did not decrease, whereas proteins and nucleic acids were degraded at the late stage of senescence. In unpollinated, detached flowers, nutrient remobilization may be reduced from that in flowers that are still attached to the plant during senescence [1]. An investigation using attached flowers would clarify the role of PCD in nutrient remobilization in epidermal and mesophyll cells.

## Conclusion

This study demonstrated a time lag of PCD progression between mesophyll and epidermal cells during flower life in petals of *Lilium* cv. Yelloween. The early onset of PCD only occurred in some mesophyll cells, and 22.8% of the mesophyll cells were lost from day 0 to day 3 after anthesis. Early expression of KDEL-type cysteine proteinase (*LoCYP*) and S1/P1 nuclease (*LoNUC*) genes may be involved in the early onset of PCD in mesophyll cells. The early onset of PCD in mesophyll cells appears to play an important role in nutrient remobilization from senescent petals to other plant parts. Five days after anthesis, signs of the late onset of PCD were observed in the mesophyll cells. Beginning on day 5, PCD rapidly progressed in the epidermis. The results of gene expression analysis suggest that *LoCYP*, *LoNUC*, and SAG12 cysteine proteinase gene *LoSAG12* are involved in the late onset of PCD in epidermal cells. The contributions of *LoSAG12* appeared to be higher in epidermal cells than in mesophyll cells.

## Supporting Information

S1 Fig Changes in dry weight of epidermal and mesophyll cells per petal. Central bundles and the bottom part of each petal were excluded from samples. Means with different letters are significantly different (Tukey test, *p* < 0.05). Each data point represents the mean ± S.E. (n = 3).
(TIF)

S2 Fig Staining sections with Evans Blue dye. Black arrows, partially stained epidermal section. Bars, 10 mm.
(TIF)

S3 Fig Alignment of the deduced amino acid sequences of SAG12 cysteine proteinase enzymes. The sequences of *LoSAG12* are compared with the sequences of *SAG12* (*In33*) from *I*. *nil* (accession AB267829), *CP1* from *N*. *tabacum* (accession AY881011), *A*. *thaliana* (accession AAC49135), and *BnSAG12-1* from *Brassica napus* (accession AAD53011). The ERFNIN motif within the prosequence, amino acids belonging to the catalytic triad (Cys_140_- His_277_- Asn_298_), and other amino acids important for catalysis (Phe_267_, Trp_300_, Trp_304_, Gln_134_) are shown in red. Cysteine residues involved in disulfide bridges are shown in blue.
(TIF)

S4 Fig Alignment of the deduced amino acid sequences of KDEL-tailed cysteine proteinase enzymes. The sequences of *LoCYP* are compared with the sequences of *CEP3* from *A*. *thaliana* (accession NP_566901), *CP2* from *N*. *tabacum* (accession AY881010), and *CYP* from *L*. *longiflorum* (accession HF968474). The ERFNIN motif within the prosequence, amino acids belonging to the catalytic triad (Cys_154_-His_289_- Asn_310_), and another amino acid (Gln_148_) important for catalysis are in red. Cysteine residues involved in disulfide bridges are shown in blue and the C-terminal KDEL is shown in green.
(TIF)

S5 Fig Alignment of the deduced amino acid sequences of vacuolar processing enzyme (VPE) cysteine proteinase enzymes. The sequences of *LoVPE* are compared with the sequences of αVPE (accession D61393), βVPE (accession D61394), γVPE (accession BAA18924) and δ*VPE* (accession AF521661) from *A*.*thaliana*. Amino acids belonging to the catalytic pocket (Cys-_204_ and His-_162_) are shown in red. The C-terminal GFSA motif is shown in green.
(TIF)

S6 Fig Phylogenetic analysis of vacuolar processing enzymes (VPEs). VPEs cloned from *A*. *thaliana* (At), *N*. *tabacum (Nt)*, *Hordeum vulgare*, *(Hv) L*. *longiflorum (Ll)*, *I*. *nil (In)*, *Dianthus caryophyllus (Dc)*, *Narcissus pseudonarcissus (Np)*, and *Theobroma cacao (Tc)*. *LoVPE* is boxed. Sequence alignment was performed with ClustalW2.
(TIF)

S7 Fig Alignment of the deduced amino acid sequences of S1/P1 type nuclease enzymes. The sequences of *LoNUC* are compared with those of SA6 from *Hemerocallis* (accession AF082031), *BFN1* from *A*. *thaliana* (accession NM_100991.2), *ZEN1* from *Zinnia elegans* (accession AB003131), S1 from *Aspergillus oryzae* (accession D45902), and *P1 Penicillium chrysogenum* (accession XM_002557445). The active site residues involved in the binding of zinc atoms are shown in red. Cysteine residues involved in disulfide bridges are shown in blue.
(TIF)

S1 Table Sequences of primers used in real-time reverse transcription polymerase chain reaction. (DOCX)
