# Supplementary figures and images for: Programmed Cell Death Progresses Differentially in Epidermal and Mesophyll Cells of Lily Petals
Source: PLoS One. 2015 Nov 25;10(11):e0143502. doi: 10.1371/journal.pone.0143502 (PMC4659684; doi:10.1371/journal.pone.0143502)

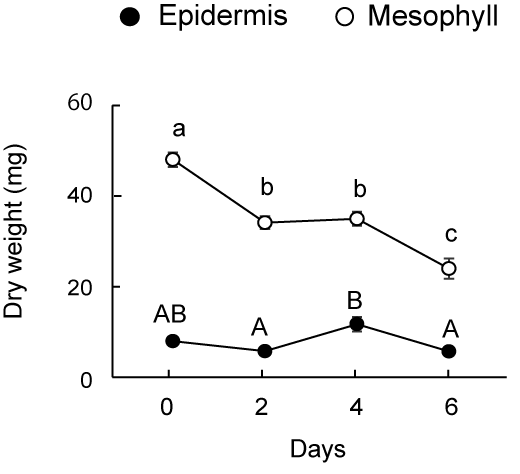

Supplement: S1 Fig — Central bundles and the bottom part of each petal were excluded from samples. Means with different letters are significantly different (Tukey test, p < 0.05). Each data point represents the mean ± S.E. (n = 3). (TIF) [file pone.0143502.s001.tif]

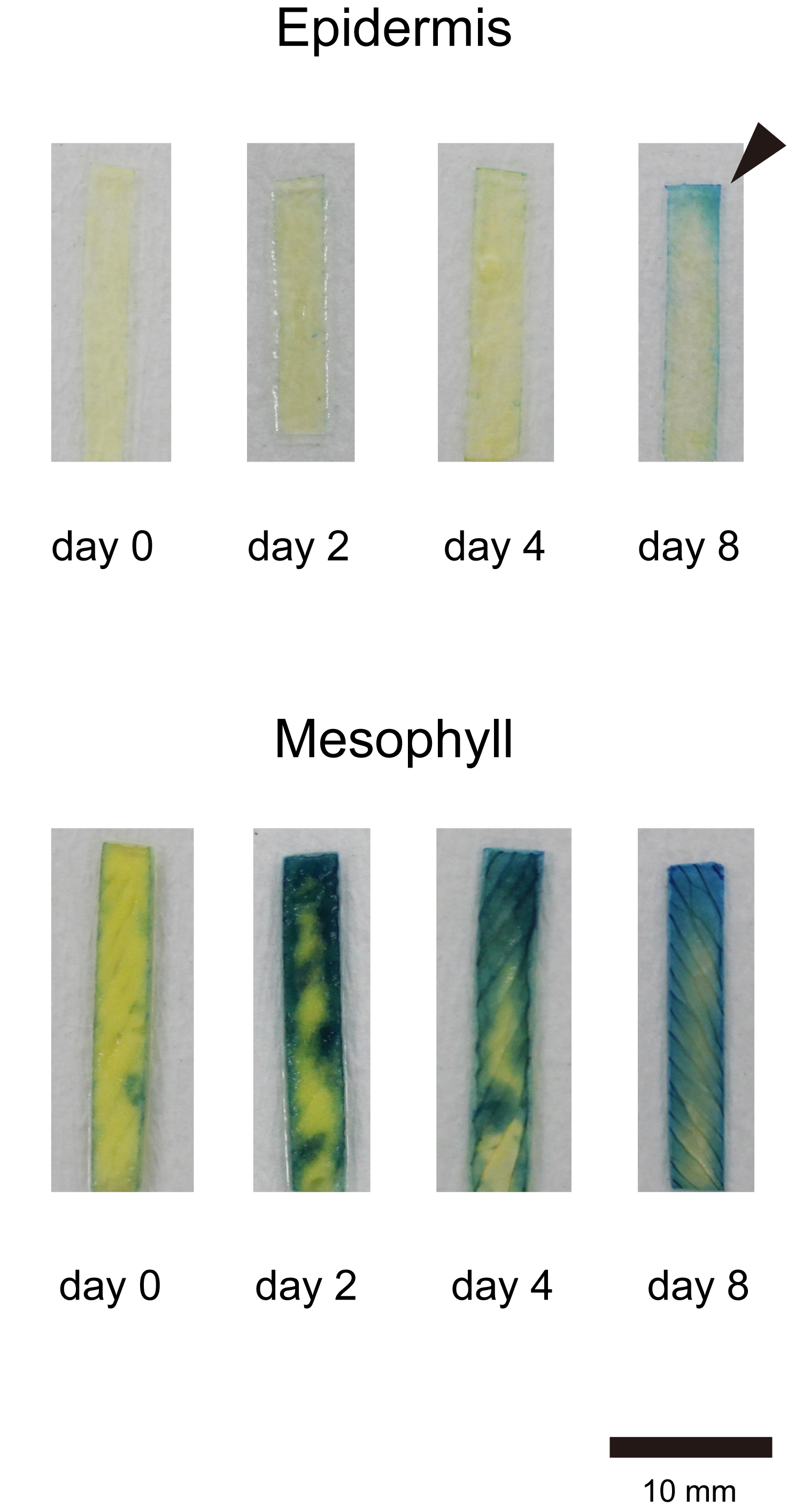

Supplement: S2 Fig — Black arrows, partially stained epidermal section. Bars, 10 mm. (TIF) [file pone.0143502.s002.tif]

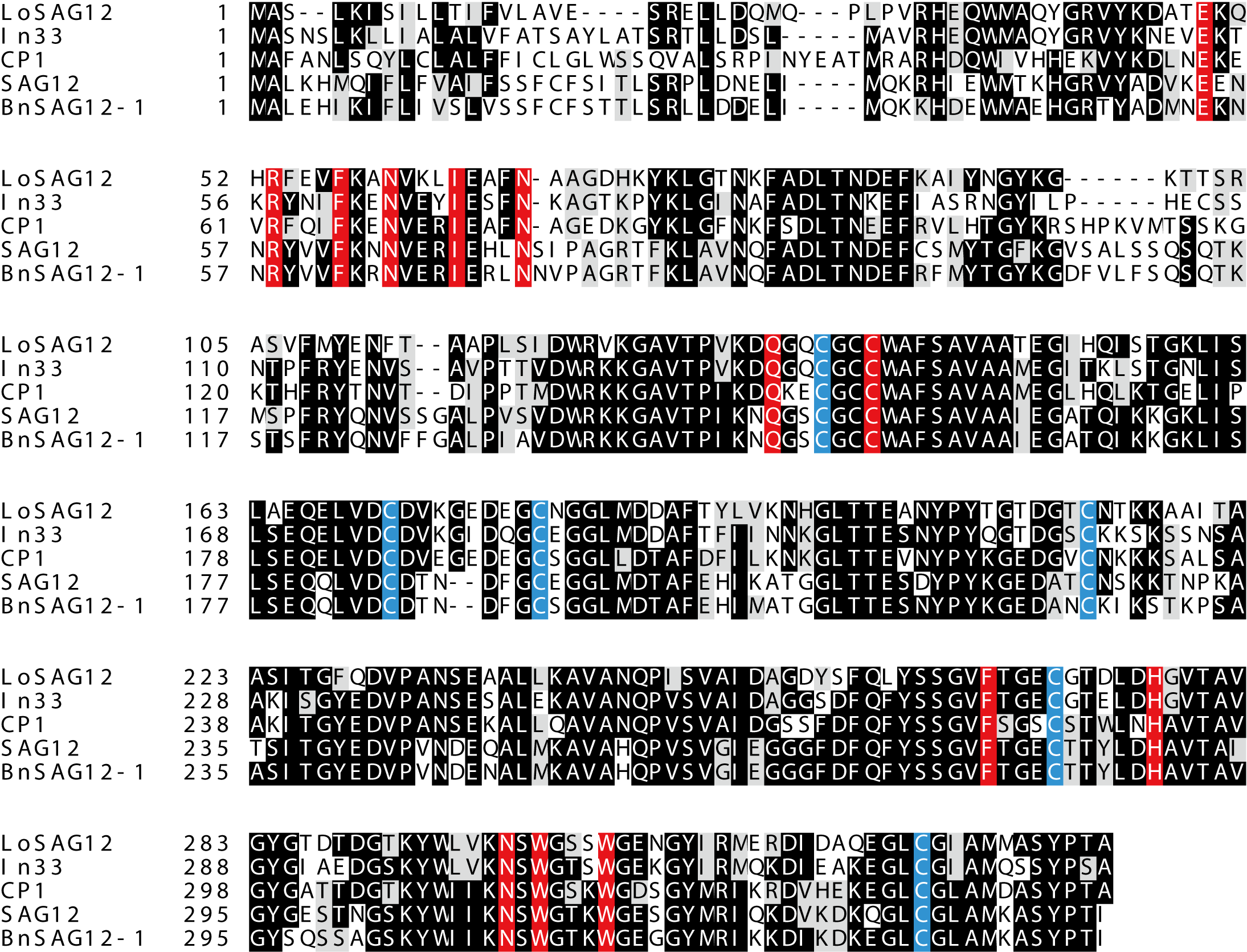

Supplement: S3 Fig — The sequences of LoSAG12 are compared with the sequences of SAG12 (In33) from I. nil (accession AB267829), CP1 from N. tabacum (accession AY881011), A. thaliana (accession AAC49135), and BnSAG12-1 from Brassica napus (accession AAD53011). The ERFNIN motif within the prosequence, amino acids belonging to the catalytic triad (Cys140- His277- Asn298), and other amino acids important for catalysis (Phe267, Trp300, Trp304, Gln134) are shown in red. Cysteine residues involved in disulfide bridges are shown in blue. (TIF) [file pone.0143502.s003.tif]

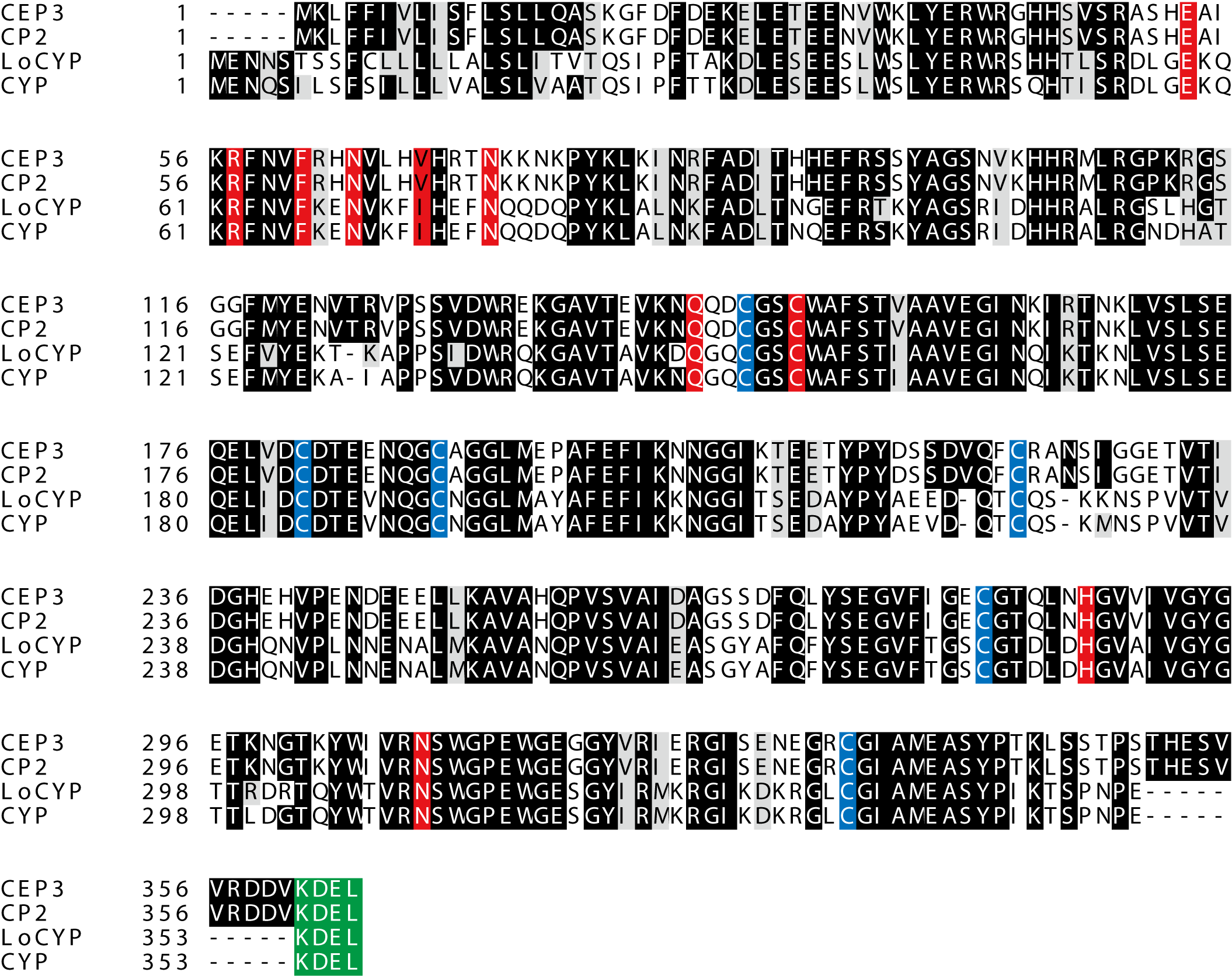

Supplement: S4 Fig — The sequences of LoCYP are compared with the sequences of CEP3 from A. thaliana (accession NP_566901), CP2 from N. tabacum (accession AY881010), and CYP from L. longiflorum (accession HF968474). The ERFNIN motif within the prosequence, amino acids belonging to the catalytic triad (Cys154-His289- Asn310), and another amino acid (Gln148) important for catalysis are in red. Cysteine residues involved in disulfide bridges are shown in blue and the C-terminal KDEL is shown in green. (TIF) [file pone.0143502.s004.tif]

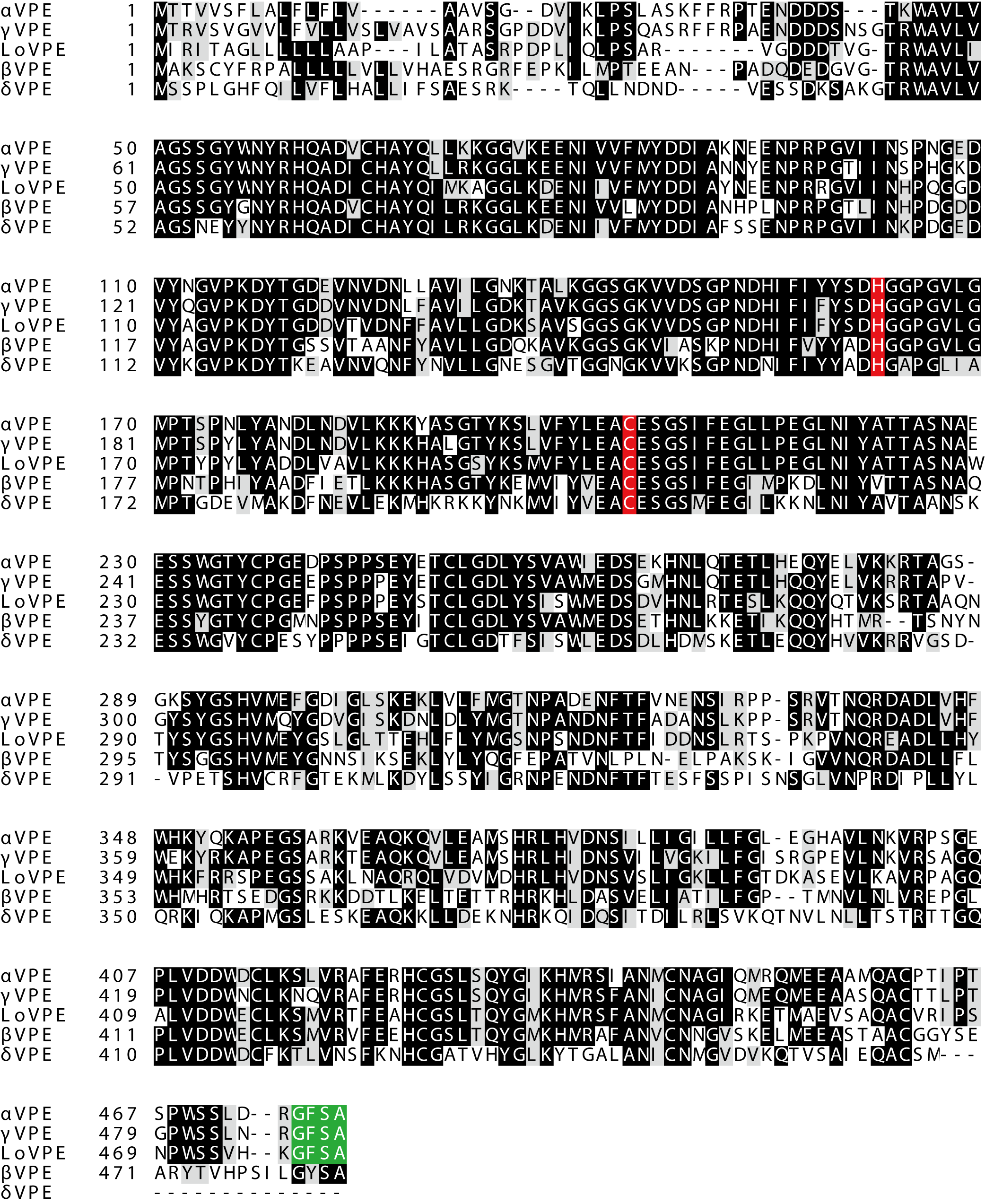

Supplement: S5 Fig — The sequences of LoVPE are compared with the sequences of αVPE (accession D61393), βVPE (accession D61394), γVPE (accession BAA18924) and δVPE (accession AF521661) from A.thaliana. Amino acids belonging to the catalytic pocket (Cys-204 and His-162) are shown in red. The C-terminal GFSA motif is shown in green. (TIF) [file pone.0143502.s005.tif]

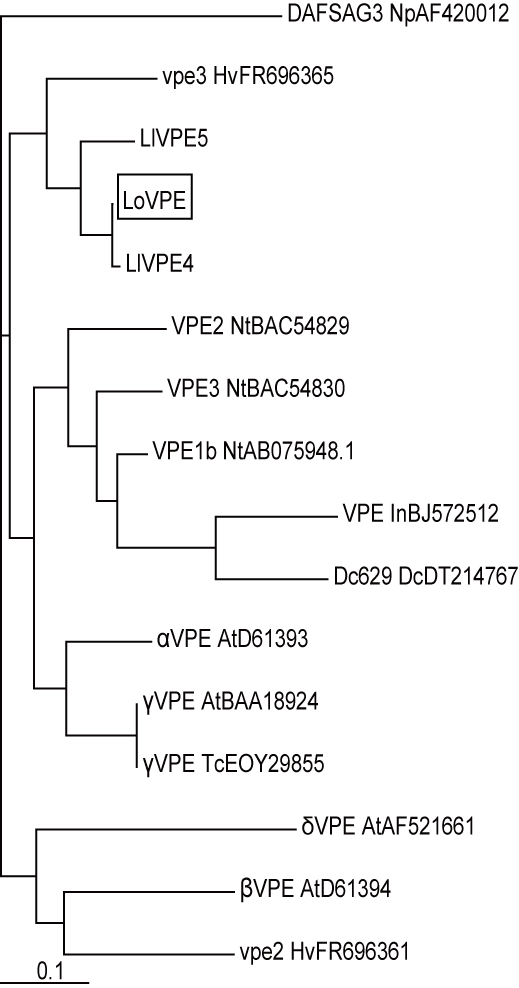

Supplement: S6 Fig — VPEs cloned from A. thaliana (At), N. tabacum (Nt), Hordeum vulgare, (Hv) L. longiflorum (Ll), I. nil (In), Dianthus caryophyllus (Dc), Narcissus pseudonarcissus (Np), and Theobroma cacao (Tc). LoVPE is boxed. Sequence alignment was performed with ClustalW2. (TIF) [file pone.0143502.s006.tif]

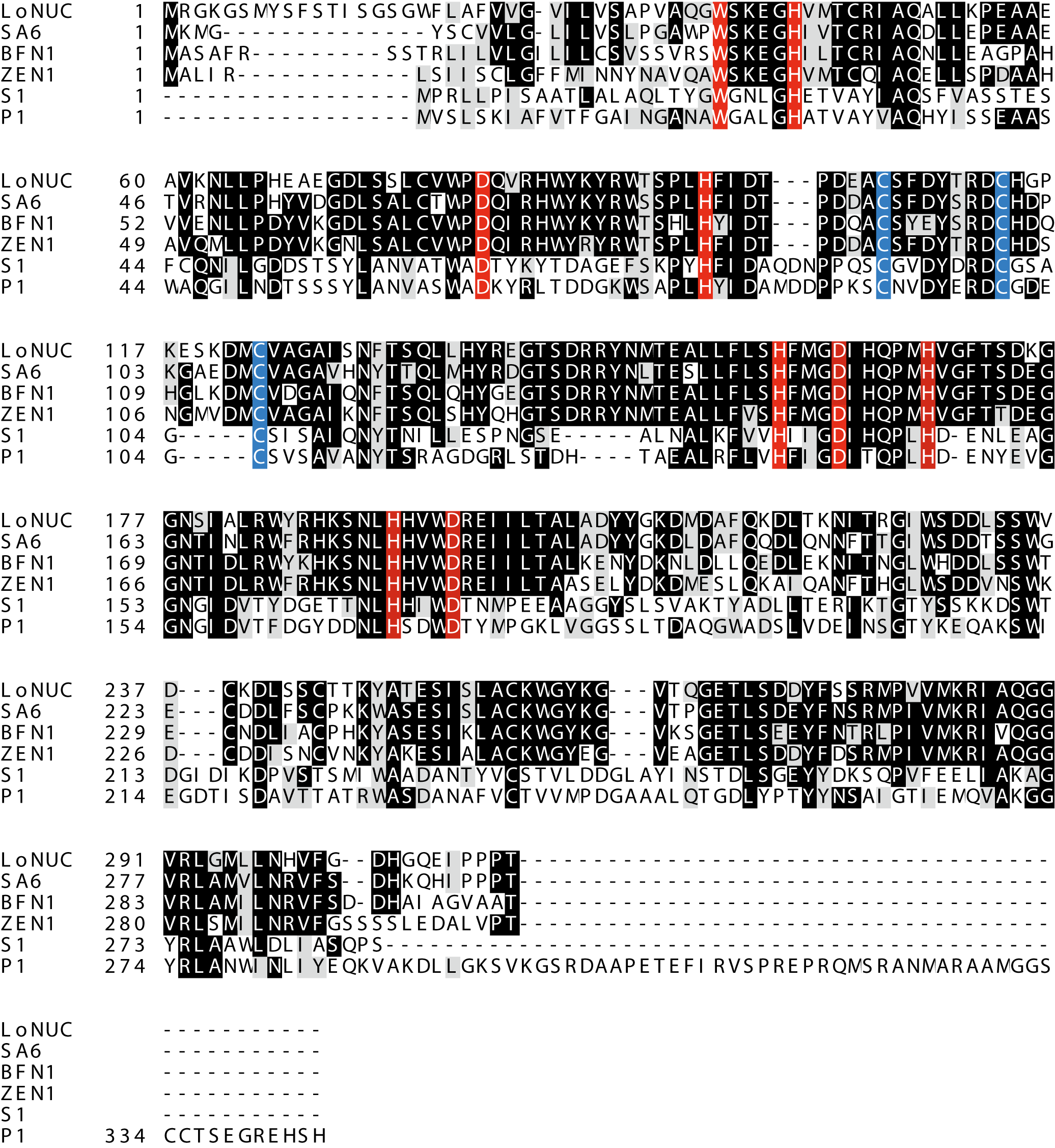

Supplement: S7 Fig — The sequences of LoNUC are compared with those of SA6 from Hemerocallis (accession AF082031), BFN1 from A. thaliana (accession NM_100991.2), ZEN1 from Zinnia elegans (accession AB003131), S1 from Aspergillus oryzae (accession D45902), and P1 Penicillium chrysogenum (accession XM_002557445). The active site residues involved in the binding of zinc atoms are shown in red. Cysteine residues involved in disulfide bridges are shown in blue. (TIF) [file pone.0143502.s007.tif]
