# Supplementary material for: Programmed Cell Death Progresses Differentially in Epidermal and Mesophyll Cells of Lily Petals
Source: PLoS One. 2015 Nov 25;10(11):e0143502. doi: 10.1371/journal.pone.0143502 (PMC4659684; doi:10.1371/journal.pone.0143502)
Supplement: S1 Table — (DOCX) [file pone.0143502.s008.docx]

S1 Table. Sequences of primers used in real-time reverse transcription polymerase chain reaction

| Gene | Forward (5′ to 3′) | Reverse (5′ to 3′) |
| --- | --- | --- |
| *LoSAG12* | GGCATCATACCCAACTGCTT | AGCAAGCACGAAGGAAGCTA |
| *LoCYP* | ATGGAGGCGTCCTACCCTAT | TGCATGACATGAAACACTTTACA |
| *LoVPE* | GTGTAAGGATCCCGTCGAAC | TCGAACTTCATGCCTCCTATC |
| *LoNUC* | CCAACCTGAAGAGGGTTGTC | GGCTGGCACTGCTATTTCAT |
| *LoUBQ* | TGCCTCCTGTCTGTTACCTTG | TTCAACCACATTAGACCACCA |
